# Targeting of embryonic annexin A2 expressed on ovarian and breast cancer by the novel monoclonal antibody 2448

**DOI:** 10.18632/oncotarget.24152

**Published:** 2018-01-10

**Authors:** Simeon Cua, Heng Liang Tan, Wey Jia Fong, Angela Chin, Ally Lau, Vanessa Ding, Zhiwei Song, Yuansheng Yang, Andre Choo

**Affiliations:** ^1^ Stem Cells 1 Group, Bioprocessing Technology Institute (BTI), Agency for Science, Technology and Research (A*STAR), Singapore 138668, Singapore; ^2^ Proteomics Group, Bioprocessing Technology Institute (BTI), Agency for Science, Technology and Research (A*STAR), Singapore 138668, Singapore; ^3^ Expression Engineering 1 Group, Bioprocessing Technology Institute (BTI), Agency for Science, Technology and Research (A*STAR), Singapore 138668, Singapore; ^4^ Animal Cell Technology 1 Group, Bioprocessing Technology Institute (BTI), Agency for Science, Technology and Research (A*STAR), Singapore 138668, Singapore; ^5^ Department of Biomedical Engineering, Faculty of Engineering, National University of Singapore (NUS), Singapore 117575, Singapore

**Keywords:** annexin a2, antibody, antibody-dependent cell-mediated cytotoxicity, antibody drug conjugate, new targets

## Abstract

Monoclonal antibodies (mAbs) play an increasingly important role in cancer therapy. To address the wide heterogeneity of the disease, the identification of novel antigen targets and the development of mAbs against them are needed. Our lab previously generated a panel of mAbs against human embryonic stem cells (hESC) using a whole cell immunization approach in mice. These mAbs can potentially target oncofetal antigens and be repurposed for antibody or antibody drug conjugate (ADC) therapy. From this panel, the novel IgG1 2448 was found to bind surface antigens on hESC and multiple cancer cell lines. Here, we show 2448 targets a unique glycan epitope on annexin A2 (ANXA2) and can potentially monitor the Epithelial-Mesenchymal Transition (EMT) in ovarian and breast cancer. To evaluate 2448 as a potential drug, 2448 was engineered and expressed as a chimeric IgG1. Chimeric 2448 (ch2448) demonstrated efficient and specific killing when conjugated to cytotoxic payloads as an ADC. In addition, ch2448 elicited potent antibody-dependent cell-mediated cytotoxicity (ADCC) activity *in vitro* and *in vivo*. Further engineering of ch2448 to remove fucose in the Fc domain enhanced ADCC. Overall, these findings indicate that embryonic ANXA2 is an attractive target and suggest that ch2448 is a promising candidate for further therapeutic development.

## INTRODUCTION

Monoclonal antibodies (mAbs) have emerged as an important class of biologics for targeted cancer therapy. As a targeted drug, mAbs can bind to antigens that are specifically expressed on the surface of cancer cells. Additionally, they can exhibit diverse modes of action: direct tumor cell-killing, modulation of a host immune system and delivery of toxic agents as an antibody-drug conjugate (ADC). To date, over twenty-five antibody-based therapies have been approved for cancer. However, these antibodies target a limited number of tumor antigens, such as CD20, ERBB2 and EGFR. To address the molecular heterogeneity of cancer, the discovery and exploitation of new antigen targets is needed.

Tumor antigens are upregulated or transiently expressed in non-cancerous cells during particular stages of differentiation and development. For example, early stem cell surface markers like Tumor Rejection Antigen-1-60 (TRA-1-60) and the Stage Specific Embryonic Antigen-3 (SSEA-3) were originally raised against embryonal carcinoma cells and later identified in breast and prostate cancer subpopulations [1]. Antigens that are associated with embryonic and fetal development such as these can be classified as oncofetal antigens and include commonly-used cancer serum biomarkers (eg. alpha-fetoprotein (AFP) for hepatocellular cancer and germ cell tumors, carcinoembryonic antigen (CEA) for colorectal cancer, sialylated Lewis A antigen (CA19-9) for pancreatic cancer and Mucin 16 (CA-125) for ovarian cancer. These biomarkers are generally used to monitor cancer disease progression and to a lesser extent, as a diagnostic or prognostic marker. More recently, oncofetal antigens have been reported as promising targets for cancer vaccines [2] and antibody-based therapies [3].

Human embryonic stem cells (hESC) represents a possible source of oncofetal antigens [1, 4]. To discover new possible oncofetal targets, we took advantage of a panel of mAbs that was previously generated using intact hESC as the immunogen [5, 6]. This approach allows for targeting native conformational epitopes including post-translational modifications like glycosylation [7]. From this panel, our group has reported on mAbs targeting known oncofetal antigens epithelial cell adhesion molecule (EpCAM, CD326) [8] and podocalyxin-like protein-1 (PODXL-1) [9].

Here, we report on 2448, a mouse IgG_1_ from the same panel of mAbs. Apart from the ability to select for undifferentiated hESC [6], 2448 was found to also bind on the surface of epithelial ovarian and breast cancer cells; and the antigen target of 2448 was identified as a glycosylated epitope on annexin A2 (ANXA2). More importantly, a chimerized 2448 (ch2448) was found to kill cancers via both an antibody drug conjugate (ADC) and antibody-dependent cell-mediated cytotoxicity (ADCC) route in cell-based assays and mouse xenograft models.

## RESULTS

### Antibody 2448 targets cancer cells expressing an epithelial phenotype of EMT

Flow cytometry was performed to assess the ability of mAbs to recognize cell surface antigens. Antibody 2448 was found to specifically bind to HES-3 (hESC) and 2102Ep (embryonal carcinoma cells) but not on normal adult cell lines: HFF (human foreskin fibroblast), IMR90 (lung fetal fibroblast) and HEK293 (human embryonal kidney) (Supplementary Table 1). To determine the binding profile against non-germ cell cancers, the screening was expanded to a panel of thirty-three human ovarian and breast carcinoma cells (Table 1). Homogenous binding was observed with binding percentages of cell populations ranging from 0% to over 80%. Greater than 60% binding was observed in eight serous ovarian carcinoma lines and ten breast carcinoma lines. Only slight positive binding was observed on MCF10A (breast epithelial cells) and no binding was observed on IOSE523 (immortalized ovarian surface epithelium cells). Furthermore, preliminary immunohistochemistry (IHC) data demonstrated positive binding of 2448 on a subset of ovarian and breast tumors compared to uninvolved normal tissue in multi-tumor tissue microarrays (Supplementary Figure 2). Results suggest that 2448 specifically targets cell surface antigens on a subpopulation of ovarian and breast cancers.

**Table 1:** Binding of antibody 2448 on the surface of ovarian and breast cell lines

Ovarian		mAb clone	Breast		mAb clone
EMT Category^†^	Cell line	2448^º^	EMT Category^†^	Cell line	2448^º^
E	CaOV3	+++	E	BT474	++++
E	OVCAR3	+++	E	CAMA1	++++
E	OVCAR8	++++	E	HCC2218	++++
E	OV90	+++	E	MDA453	++++
E	IGROV1	++++	E	MCF7	++++
IE	OV17R	+	E	SKBR3	++++
IE	OVCA432	++++	E	T47D	++++
IE	OVCA433	+++	IE	BT20	++++
IM	CH1	+	IE	HCC1937	++++
IM	HEY	–	IM	HCC1954	++++
IM	HEYC2	–	M	MCF10A	++
IM	SKOV3	+++	M	MDA-MB-231	–
IM	SKOV3(D10)	+	M	BT549	–
M	A2780	–	M	HS578T	++
M	COLO720E	–	U	184B5	++++
M	HEYA8	–	U	HCC1395	–
M	OVCAR10	–			
M	TOV112	–			
U	IOSE523	–			

Supernatant from hybridoma clones of 2448 was screened by flow cytometry on eighteen ovarian tumor cell lines and one immortalized ovarian surface epithelium cell line, IOSE523 and fourteen breast tumor cell lines and one immortalized mam mary epithelial cell line, MCF10A. Overall, stronger binding was observed on cell lines with an Epithelial or Intermediate Epithelial phenotype. Binding intensity was graded according to binding percentages after marker gating with a 2.0–2.5% cut-off from the negative control: “–” < 10% binding, “+” = 10–40% binding, “++” = 40–60%, “+++” = 60–80% and “++++” > 80%.

^†^Cell lines were classified according to an Epithelial-Mesenchymal Transition phenotype based on work done by Huang *et al*. [13] and Akalay *et al*. [14]: “E” = Epithelial, “IE” = Intermediate Epithelial, “IM” = Intermediate Mesenchymal, “M” = Mesenchymal and “U” = Not Classified. Cell lines 184B5, HCC1395 and IOSE523 were not classified (U).

The EMT process is closely associated with the carcinoma progression as well as embryogenesis and the differentiation of hESC [11, 12]. Therefore, to investigate if 2448 was binding to a particular stage of EMT, cell lines were classified based on EMT scoring [13, 14], namely, epithelial (E), intermediate-epithelial (IE), intermediate-mesenchymal (IM) and mesenchymal (M). The anti-EpCAM, mAb 8, was used as a positive control for the “E” and “IE” phenotype [8].

Interestingly, 2448 shared a similar binding profile for both ovarian and breast cancer cells (Table 1). Strong to moderate binding was observed on seventeen cell lines classified as “E” or “IE”. Weak or no binding was observed on fourteen cell lines classified as “IM” or “M”. Overall, stronger binding was observed on cells with an epithelial phenotype, suggesting that 2448 preferentially targets cancers with an epithelial phenotype. Notable exceptions include the “IM” lines SKOV3, CH1 and HS578T cell lines.

**Figure 1:**
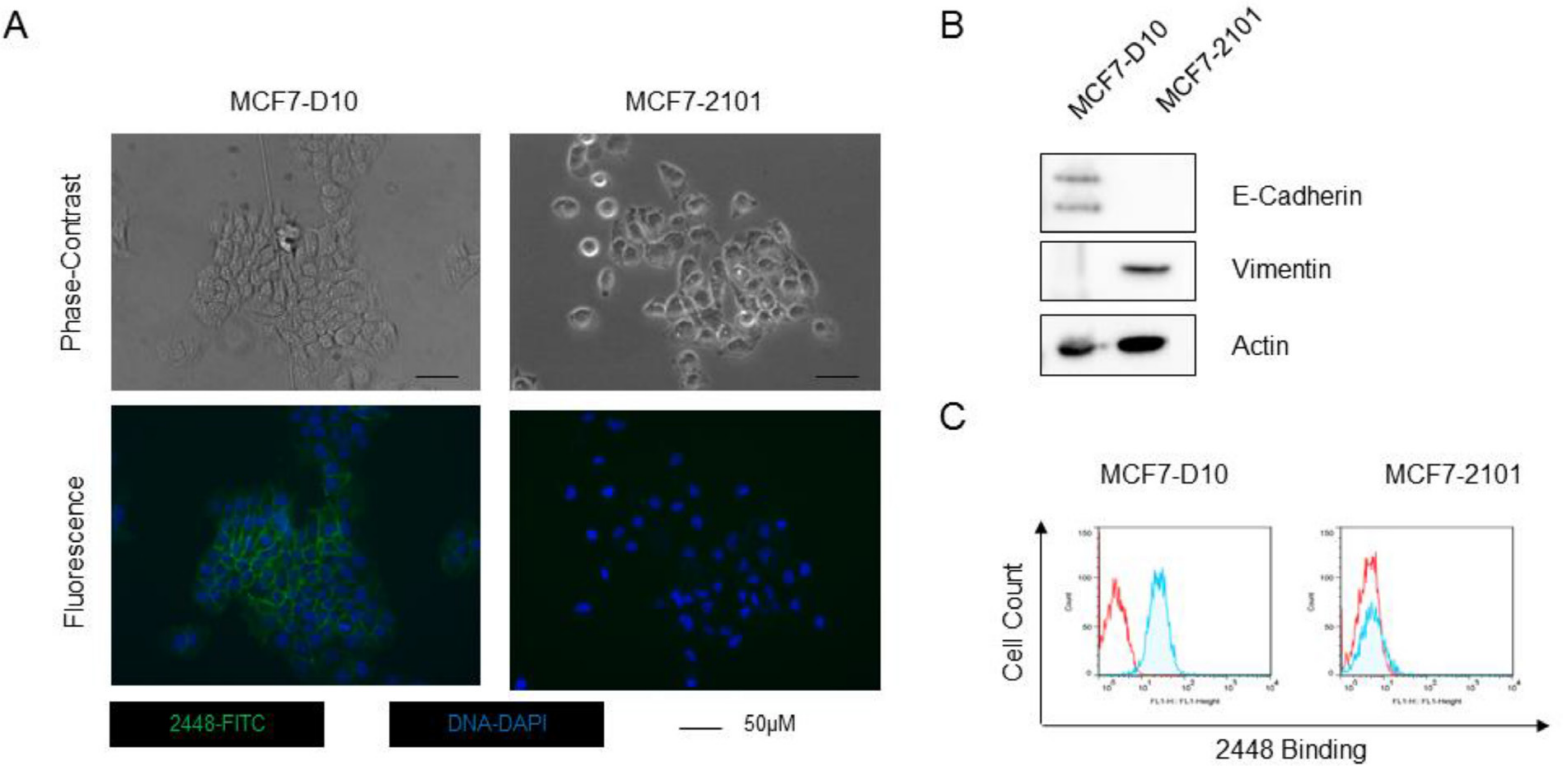
Binding of 2448 on two isogenic breast cancer cell line The isogenic breast cancer cell lines (MCF7-D10 and MCF7-2101) represent epithelial and mesenchymal phenotypes of EMT. (**A**) MCF-D10 and MCF7-2101 isogenic cell lines were stained with 2448 and visualized by epifluorescence microscopy. Membranous binding of 2448 was observed on MCF7-D10 cells but not on the isogenic MCF7-2101 cells. The loss of binding on MCF7-2101 corresponded to EMT-like morphological changes. MCF7-D10 are more epithelial with cuboidal or “cobblestone-like” cells whereas MCF7-2101 cells are more mesenchymal-like (ie. more isolated and elongated). (**B**) High levels of E-cadherin and low levels of the vimentin were expressed in MCF7-D10 cells versus MCF72101 cells. (**C**) Flow cytometry confirmed binding of 2448 on live MCF7-D10 cells, but not live MCF7-2101 cells. Positive binding is indicated by a rightward shift in fluorescence intensity (shaded) of histogram.

To confirm that 2448 specially targets cells with epithelial phenotype of EMT, 2448 was also screened against the MCF7-2101 cell line, which was derived by the treatment of MCF7-D10 with TNF-alpha, a known inducer of EMT [14]. Compared to epithelial MCF7-D10, isogenic MCF7-2101 cells expressed a mesenchymal phenotype of EMT. Morphological differences such as cell scattering and elongated fibroblastic geometry were observed by phase-contrast microscopy (Figure 1A) and confirmed by a lower expression of the canonical epithelial marker, E-Cadherin and higher expression of the mesenchymal marker, vimentin (Figure 1B). By flow cytometry, 2448 was found to only bind live MCF-D10 but not MCF7-2101 (Figure 1C). Accordingly, immunostaining revealed binding of 2448 on the membranes and tight junctions of live MCF7-D10 cells (Figure 1A).

### Antibody 2448 targets a unique glycoprotein epitope of Annexin A2

**Figure 2:**
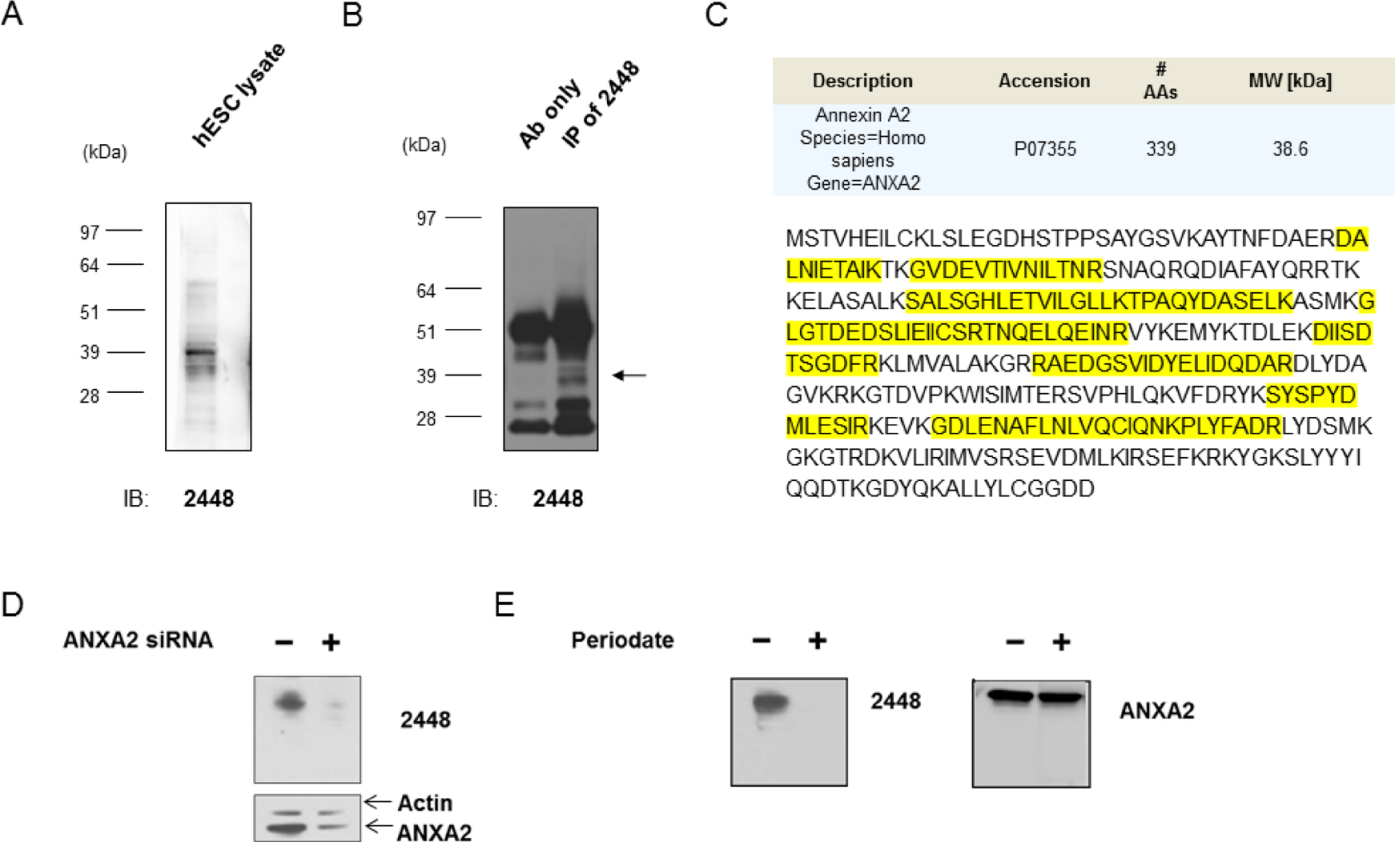
Identification of annexin A2 as the antigen target of 2448 Immunoprecipitation (IP) of lysate from IGROV1 ovarian cancer cells was done with 2448-coupled Protein G—beads. IP with 2448 enriched for unique antigen bands around 39 kDa (←) on Western blot. The corresponding bands on parallel silver stained gels were excised and sent for liquid chromatography-tandem mass spectrometry (LC-MS/MS). A search against protein databases revealed Annexin A2 as a top match for multiple rounds of IP. (**C**) Peptide coverage (as highlighted) was found to span across the amino acid sequence of ANXA2. (**D**) Antigen recognition of 2448 was abolished after cells were transfected with validated ANXA2-specific siRNA (versus scramble siRNA as a control). Loss of binding corresponded to a decrease in ANXA2 expression using a commercial anti-ANXA2 mAb. Results were normalised against beta-actin expression. (**E**) Antibody 2448 targeted a glycosylated epitope. A mild periodate treatment abolished binding of 2448 on Western blot. No loss of binding was observed using a commercial anti-ANXA2 mAb targeting ANXA2 peptide. Abbreviations: Ab = antibody AA = amino acid, ANXA2 = annexin A2, MW = molecular weight.

Next, the antigen target of 2448 was characterized on Western blot. Similar antigen bands were detected on lysate from HES-3 (Figure 2A) and various ovarian and breast cancer cells (Figure 2B and Supplementary Figure 3B) at approximately 36 to 40 kDa. It is interesting to note that the antigen bands had a similar smeary appearance between these different cell lines. Immunoprecipitation (IP) was carried out to enrich for the antigen of interest in hESC and IGROV1 ovarian cancer cells (Figure 2B). Corresponding bands on a silver stained gel were excised and analyzed by liquid chromatography tandem mass spectrometry (LC-MS/MS). Annexin A2 (ANXA2; Accession No. P07355) was identified as the putative target for 2448. Multiple rounds of IP and LC-MS/MS demonstrated that peptide coverage spanned across the amino acid sequence of ANXA2 (Figure 2C).

To validate the antigen target, a transient knockdown of ANXA2 was performed using IGROV1 cells (Figure 2D). After transfection of cells with ANXA2-targeting siRNA, partial knockdown of ANXA2 was observed by a decrease (40% to 60%) in antibody detection using commercial anti-ANXA2 mAb. Concurrently, a loss of antigen recognition by 2448 was observed on Western blot. Taken together, the results confirmed ANXA2 as the antigen target of 2448.

As ANXA2 is known to undergo posttranslational modification, such as glycosylation [15], binding of 2448 to glycans was investigated. Sodium *meta*-periodate was used as a mild oxidative treatment on Western blot. Periodate treatment modified vicinal hydroxyl groups of saccharides on the antigen, thereby disrupting antibody interaction that involved glycosylation at its recognition site. After modifications of glycans, antigen recognition by 2448 was completely abolished. In contrast, binding of a commercial anti-ANXA2 mAb, which was generated against a recombinant ANXA2 peptide (of amino acids 123–328), was not sensitive to treatment (Figure 2E). Results suggested that 2448 was binding to a unique glycan epitope on ANXA2.

To further investigate, 2448 was compared with the commercial anti-ANXA2 mAb using flow cytometry and Western blot analysis. Unlike 2448, the commercial anti-ANXA2 mAb did not bind on live IGROV1 cells. Binding was present only after cell fixation and permeabilization, suggesting commercial mAb targeted an intracellular epitope of ANXA2 (Supplementary Figure 3A). Based on Western blot analysis, the commercial mAb also detected ANXA2 on all seven cell lines regardless of their EMT classification (Supplementary Figure 3B), whilst 2448 antigen expression was only observed on cells with an epithelial EMT phenotype (corresponding to flow cytometry data (Table 1)). Taken together, these results suggested that 2448 specifically binds to a unique glycan epitope on ANXA2 expressed on the cell surface of ovarian and breast cancer cells.

### Generation of a chimeric variant of 2448 (ch2448)

To date, most mAbs that are used in the clinic are IgG1 and chimerized if not humanized. Therefore, 2448 was chimerized by cloning the variable region of 2448 into a multi-promoter single expression vector containing human IgG_1_ constant genes [10]. This vector was then transfected into the DG44 Chinese hamster ovary (CHO) cell line for high and stable expression (>1 g/L). Peptide sequencing confirmed the variable regions of chimeric 2448 (ch2448) matched those of the parental murine 2448 (data not shown). As expected, ch2448 had similar antigen binding properties as the parental murine 2448 (Supplementary Figure 4). The chimeric 2448 was then evaluated for functional efficacy as either a naked antibody therapy or as an ADC therapy.

### Antibody 2448 rapidly internalizes and delivers cytotoxic secondary conjugates into cells

**Figure 3:**
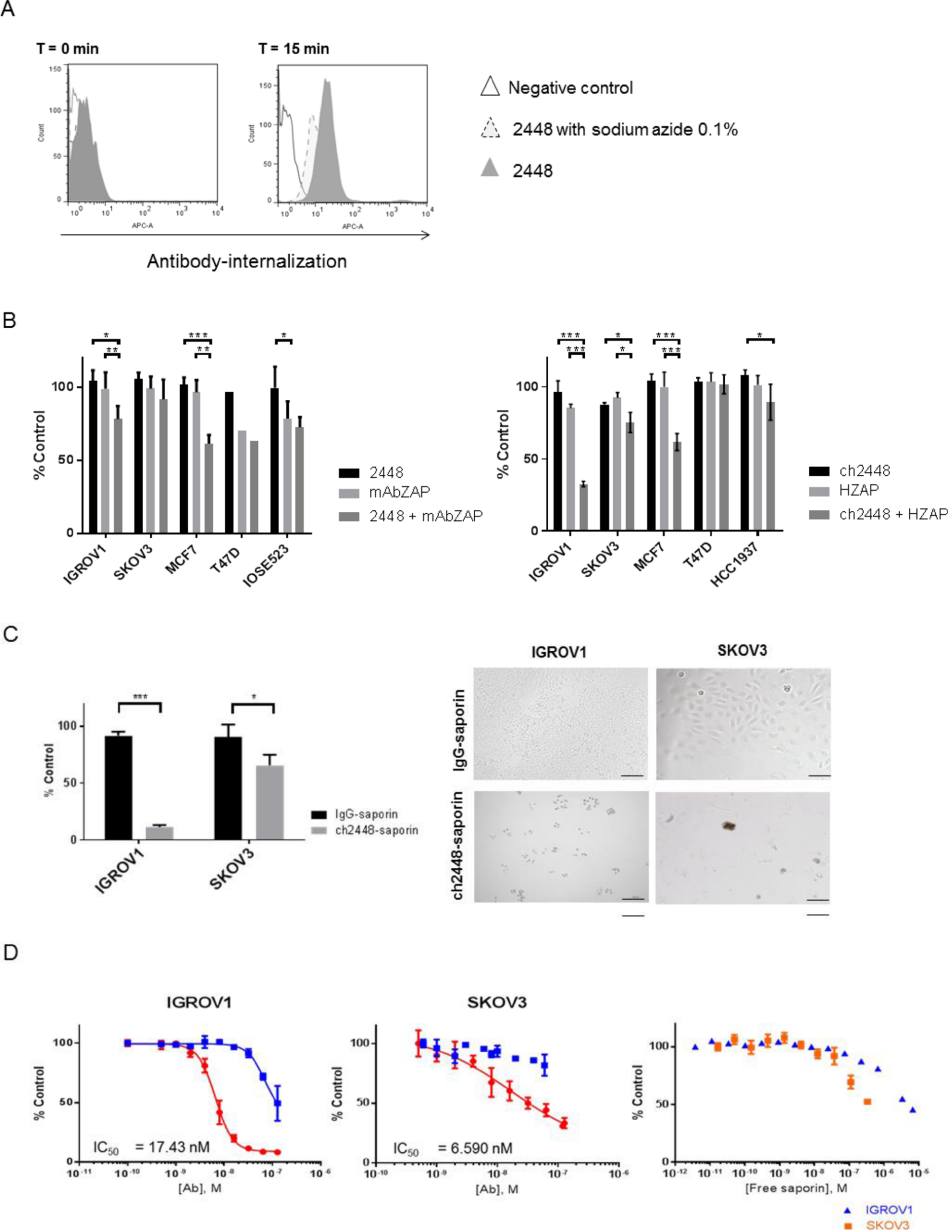
Delivery of cytotoxic payloads by 2448 and ch2448 The potential of 2448 to be developed as an ADC was assessed. (**A**) Antibody 2448 was labelled with Cypher5e dye that maximally fluoresces in low pH environments (such as endosomal and lysosomal cellular compartments). At 4° C, IGROV1 cells were pre-incubated with dye-conjugated 2448 in the presence or absence of sodium azide (or with dye alone as a negative control). At T = 0 min, the temperature of samples was raised to 37° C. At T = 15 min, 2448 internalization was observed by an increase in fluorescence intensity. Less antibody internalization was observed on cells pre-treated with sodium azide as indicated by lower fluorescence intensity. (**B**) The cytotoxic effects of mAbs in-complex with secondary saporin conjugates (ZAP) was evaluated. Primary 2448 and ch2448 were pre-mixed with anti-mouse IgG (mAbZAP) and anti-human IgG saporin conjugates (HZAP), respectively. Ovarian and breast cancer cells were incubated with mixtures, primary antibody alone (2448 or ch2448), saporin conjugate alone (mAbZAP or HZAP) or buffer as a vehicle control. Post 72 h incubation, a significant cytotoxic effect was measured in IGROV1 and MCF7 cells that were treated with mAbs in-complex with secondary conjugates compared to cells treated with primary mAb or secondary conjugate alone. (**C**) IGROV1 and SKOV3 were treated with saporin-conjugated ch2448 (ch2448-saporin) or isotype control (IgG-saporin) at 30 mM. Cell viability was measured as a percentage of cells treated with buffer alone (% control). Post 72 h incubation, an 80% and 30% decrease in viability was observed on IGROV1 and SKOV3 cells, respectively. Cells treated with ch2448-saporin had shriveled unhealthy morphologies as observed by bright-field microscopy. Results were represented as the mean ± standard deviation of three independent experiments with triplicate. (**D**) Dose-dependent cytotoxicity of ch2448-saporin was evaluated. Post 72 h incubation, greater cytotoxicity was observed for ch2448-saporin than the IgG-saporin control. IC50 values of 6.59 nM and 17.43 nM were calculated on IGROV1 and SKOV3 cells, respectively. Cytotoxicity of free saporin was observed at higher concentrations (> 0.1 µM). For (B) and (C), the relative cell viability was measured as a percentage of cells treated with buffer alone (% control). Results were represented as mean ± standard deviation of three independent experiments. Statistical significance was measured by a two-sided unpaired Student’s *t*-test (^*^p < 0.05; ^**^p < 0.01; and ^***^p < 0.001). For (D), values were means ± standard deviations of biological triplicates. Non-linear regression was performed to determine the IC50 values using GraphPad Prism 6. Similar IC50 values were observed in two independent experiments.

Antibody 2448 was first assessed for its potential to deliver a cytotoxic drug. A conventional ADC relies on efficient antibody-internalization to induce cytotoxicity. Hence the ability of 2448 to internalize into cells was evaluated. Antibody 2448 was first conjugated to a pH-sensitive dye that maximally fluoresces in the acidic endosomal and lysosomal compartments of cells. Dye conjugated-2448 was internalized into IGROV1 cells within 15 min of incubation at 37° C, as observed by an increase in fluorescence intensity compared to the negative free dye control (Figure 3A). Moreover, lower fluorescence levels were observed when cells were depleted of surface-ATP by the addition of sodium azide. Time-lapse live fluorescence microscopy was used to validate results (Supplementary Video 1). Collectively, the data suggest that upon binding to the cell surface, 2448 specifically internalizes in an energy-dependent manner (e.g. via clathrin-mediated endocytosis).

To extend these observations, an *in vitro* ADC assay was carried out using secondary conjugates of saporin (30 kDa). When released intracellularly, this plant-derived toxin acted as an rRNA N-glycosylase that inactivates the large 60S ribosomal subunit to cause apoptosis [16]. Both 2448 and ch2448 effectively delivered saporin (mAb-ZAP or HUM-ZAP) into cells, inducing cytotoxicity at similar levels (Figure 3B). The most significant decreases in cell viability (20% to 60%) were observed against the epithelial IGROV1 and MCF7 cell lines. A smaller decrease of (10% to 20%) cell viability was observed on the intermediate mesenchymal SKOV3, corresponding to weaker binding of 2448. No cytotoxicity was also observed on non-2448 binding cell lines, IOSE523 and BT549. Overall, results indicated that 2448 and ch2448 were viable as targeting agents for ADC development.

### Antibody drug conjugate ch2448-saporin induces potent cytotoxicity

To extend these observations, an ADC was created by direct conjugation of saporin to ch2448 (ch2448-saporin). As a control, an isotype chimeric IgG was also conjugated to saporin (IgG-saporin). Compared to using secondary saporin conjugates, ch2448-saporin induced greater cytotoxicity against IGROV1 and SKOV3 cells. An increase of 20–30% cytotoxicity was measured by incubating ch2448-saporin at similar molar concentrations as used in the previous ADC assay (Figure 3C). Results were visually confirmed by the presence of cell-debris and unhealthy morphology of remaining cells. Cells were also treated with ch2448-saporin at various concentrations and IC_50_ values for ch2448-saporin were estimated to be in the nanomolar range (greater than 10^–8^ M) for both IGROV1 and SKOV3 (Figure 3D). As negative controls, free saporin and the IgG-saporin conjugate reached similar levels of cytotoxicity at a greater than 10-fold concentration. Corresponding to results of the secondary conjugate assay, ch2448-saporin was more potent against IGROV1 than SKOV3. To demonstrate the sustained inhibition of cell growth, real-time monitoring of cells was also done via label-free, impedance-based cell growth analysis over a period of 120 h (Supplementary Figure 5).

### Antibody ch2448 exhibits potent antibody-dependent cell-mediated cytotoxicity (ADCC) activity which is enhanced by afucosylation (aF-ch2448)

Next, the bioactivity of naked antibody 2448 was evaluated *in vitro*. No anti-proliferative nor anti-migratory effects were observed when ch2448 was co-incubated with various ovarian and breast cancer cells (Supplementary Figure 8). Therefore, ch2448 was assessed for its ability to engage immune effector cells via ADCC.

ADCC is one important mechanism of action for therapeutic mAbs and involves interactions between antibody Fc and an Fc receptor expressed on effector cells such as NK cells and macrophages. In recent years, groups have demonstrated various ways to enhance ADCC activity, including by glycoengineering of the Fc region (as reviewed in [17]). Removal of core fucose at the conserved glycosylation site (asparagine 297) of human IgG has resulted in mAbs with enhanced ADCC activity. To enhance ADCC activity of ch2448, an afucosylated variant (aF-ch2448) was generated.

**Figure 4:**
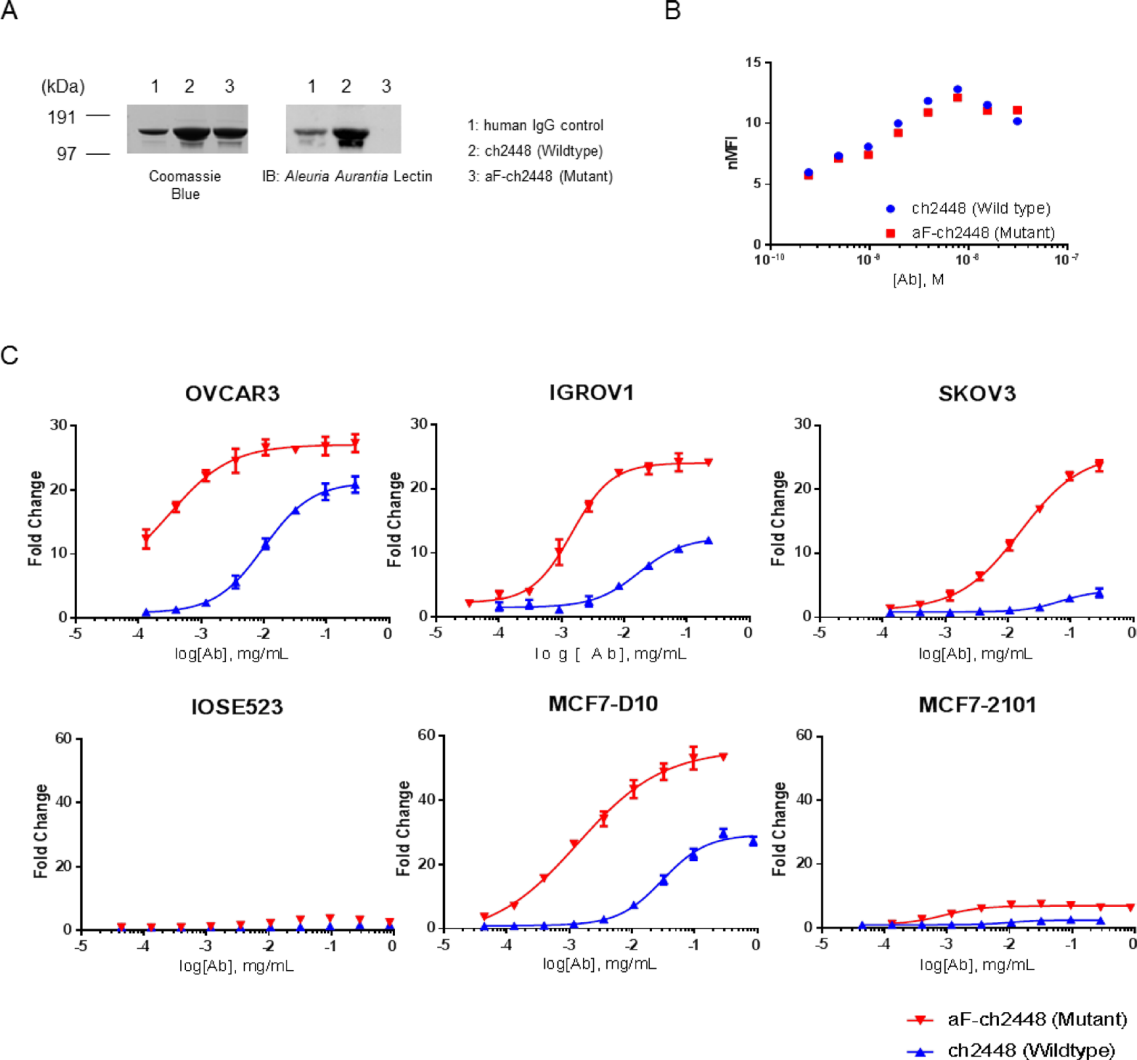
ADCC activity of afucosylated ch2448 An afucosylated variant of ch2448 (aF-ch2448) was generated. (**A**) Antibodies ch2448 and aF-ch2448 (and human IgG control) were run on SDS-PAGE in non-reducing conditions. Coomassie Blue staining of the gel showed antibodies at ∼150 kDa in size. Western blotting of samples run in-parallel showed that the *Aleuria Aurantia* Lectin was not able to recognize aF-ch2448, demonstrating the loss of core fucose. (**B**) The binding of aF-ch2448 (Mutant) retained similar specificity of ch2448 on live IGROV1 cells by flow cytometry. Cells were incubated with ch2448 or af-ch2448 at various concentrations. Binding was measured by an increase of the normalized mean fluorescence intensity (nMFI). Results were representative of three independent experiments. (**C**) ADCC activity of ch2448 and aF-ch2448 was measured on OVCAR3, IGROV1, SKOV3, MC7-D10 and MCF7-2101 cell lines. ADCC activity was measured as fold induction of the NFAT pathway. Values are means ± standard deviations of triplicates. Similar results were obtained in 3 independent experiments.

The aF-ch2448 was generated utilizing a glycoengineered mutant DG44-CHO cell line [18]. To confirm the loss of the core fucose, chimeric 2448 and aF-ch2448 were resolved by SDS-PAGE and analyzed by Western blot using fucose-binding *Aleuria Aurantia* Lectin (AAL) (Figure 4A). Wildtype (WT) ch2448 but not mutant (MT) aF-ch2448 was visible by Western blot, confirming the loss of core fucose. N-glycans of mAbs were also released and analyzed by HILIC-UPLC-QTOF experiments, confirming a drop in the percentage of fucosylation from 100% to < 1.5% (data not shown). A binding titration curve of 2448 and aF-ch2448 was also done on IGROV1 ovarian cancer cells and analyzed by flow cytometry. Both ch2448 and aF-ch2448 had similar binding profiles (Figure 4B), confirming that the loss of fucose did not alter antibody-antigen binding.

Next, the ADCC activity of ch2448 was evaluated by a gene reporter assay. The assay used engineered Jurkat cells expressing the human Fc-gamma IIIA receptor coupled to an ADCC NFAT (nuclear factor of activated T-cells) signaling pathway readout. ADCC activity of aF-ch2448 corresponded to positive binding of ch2448. After defucosylation, an increase in ADCC activity was observed on ovarian and breast cancer cells (Figure 4C). Increased efficacy was observed on OVCAR3, IGROV1 and MCF7-D10 cell lines (as indicated by the increase of the upper asymptote of dose-response curves). Maximal ADCC induction was not reached for SKOV3 at the experimental range of concentrations tested. An increase of more than 20-fold induction was observed at the highest tested concentration. No activity for either ch2448 or aF-ch2448 was observed on non-binding MCF7-2101 cells. Potency of aF-ch2448 was greater than ch2448. The EC_50_ values of aF-ch2448 were in the nanomolar range. A fold difference of approximately 20 to 25 was observed in the EC_50_ values of ch2448 compared to aF-ch2448. Among tested cell lines, OVCAR3 and SKOV3 had the lowest and highest EC_50_s (for both ch2448 and aF-ch2448), respectively. Results correlated with surface binding of 2448 (Table 1).

### Antibody ch2448 induces potent anti-tumor activity in a mouse xenograft model

**Figure 5:**
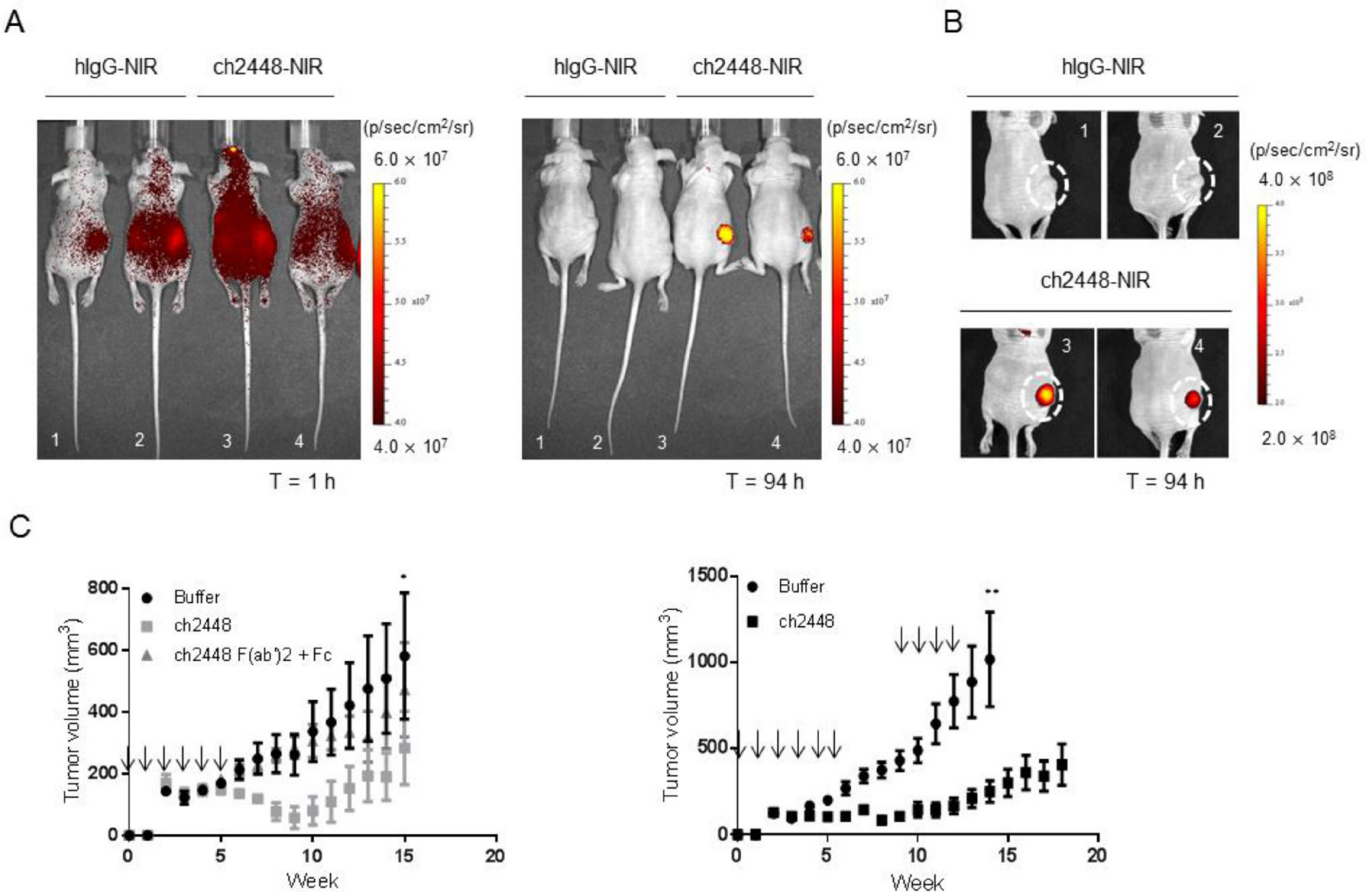
*In vivo* activity of ch2448 in a ovarian tumor xenograft mouse model (**A**) The biodistribution of ch2448 was evaluated in nude mice bearing subcutaneous IGROV1 tumor xenografts. Antibody ch2448 was conjugated to a near-infrared dye (ch2448-NIR) and administered via intraperitoneal (i.p) injection. At 1 h post-injection, ch2448-NIR was disseminated throughout the intraperitoneal cavities of mice. High levels of ch2448-NIR was observed at the subcutaneous tumor site after 96 h. Individual images of mice showed that fluorescence levels at tumor site were at least four times greater in ch2448-NIR-treated mice compared to mice treated with a dye-conjugate control (hIgG-NIR). (**B**) For mice treated with ch2448-NIR, fluorescence levels directly correlated with tumor volume. Tumor volume was measured by calipers and calculated by ½ of tumor length × width^2^. Fluorescence was measured by total flux with units of p/sec/cm^2^/sr where p = photons, sr = steradian.(**C**) Treatment with ch2448 delayed growth of an IGROV1 tumor xenograft mouse model. On day 0, nude mice were implanted subcutaneously with IGROV1 cells. The following day mice were administered ch2448 or ch2448 F(ab’)2 and Fab (or buffer alone) by i.p. injection and weekly thereafter as indicated (↓). Treatment of ch2448 delayed onset of IGROV1 tumor growth whereas no significant effects was observed with ch2448 F(ab’)2 and Fab. Results were measured by tumor volume as ((L × L × W)/2), where W (width) and L (length) are the shortest diameter, respectively. Values were represented as mean ± standard error for *n* = 5 mice. No adverse effects were observed in ch2448-treated mice. Statistical significance was measured by a two-sided unpaired Student’s *t*-test (^*^p < 0 .05; ^**^ *p* < 0.01; and ^***^p < 0.001).

So far, 2448 was shown to potentially act as an ADC or as a naked antibody that can elicit ADCC activity, *in vitro*. As saporin-based conjugates are not ideal toxins for ADC therapy, we focused on ch2448 as a naked antibody with ADCC properties. The endogenous bioactivity of ch2448 was investigated *in vivo* using an IGROV1 subcutaneous xenograft mouse model. The biodistribution of ch2448 was first assessed by directly conjugating a near infrared dye to ch2448 (ch2448-NIR) and an isotype control (hIgG-NIR). Similar degrees of labelling, 1.24 and 1.45, were measured by spectral absorbance for ch2448-NIR and hIgG-NIR, respectively. Antibody-dye conjugates were then given via intraperitoneal injection to mice bearing xenografts of similar tumor size (300–400 mm^3^). At 1 h post-injection, mice were imaged in a dorsal position (Figure 5A). Fluorescence intensity was detected throughout the bodies of all mice, especially areas surrounding the kidneys. At 96 h post-injection, mice treated with ch2448-NIR had significantly higher levels of fluorescence compared to those of the non-targeting hIgG-NIR control. Fluorescence was localized around the subcutaneous tumor region. Among the two mice treated with ch2448-NIR, higher fluorescence intensity correlated with a larger tumor volume. Results showed that ch2448 was able to target IGROV1 tumor xenografts *in vivo*.

Subsequently, the *in vivo* efficacy of ch2448 was evaluated using a pre-emptive treatment model. Nude mice were subcutaneously inoculated with IGROV1 cells and the following day, treated intraperitoneally with ch2448, either as a full length IgG_1_ or as a F(ab’)2 fragment, which does not have the ability to elicit ADCC activity (Supplementary Figure 7). Dose of IgG_1_ to F(ab’)2 fragment was given at a 1:2 molar ratio. By week 6, rapid tumor growth was observed in mice from the control group, but not in mice treated with full-length ch2448 (Figure 5C left panel). Rapid tumor proliferation of treated mice was delayed until week 10. Additionally, no significant difference was observed in control mice and those mice treated with F(ab’)2 and Fc fragment of ch2447. As no activity of F(ab’)2 was detected, results suggested that anti-tumor activity of ch2448 was based on Fc-dependent mechanisms.

To confirm these results, the study was repeated with an additional three doses of ch2448 at a higher concentration of 1 mg. Based on the previous observation of tumor growth acceleration at week 10 (Figure 5C left panel), additional treatment began at week 9. In this study, a smaller increase in tumor growth was observed compared to the buffer control group (Figure 5C right panel). At week 14, a significant difference in tumor volume between control and treatment groups was observed at week 14 (*p* < 0.001). The average tumor volume of treated group remained below 500 mm^3^. Moreover, two mice from the control group were euthanized due to tumor volumes exceeding 1500 mm^3^. Antibody treatments were generally well-tolerated in mice. Animals weighed 20–26 g at the start of treatment. No drastic loss in body weight (> 10%), side effects or abnormal behaviors were observed throughout the course of study. Overall, results showed that ch2448 targeted ovarian tumor xenografts and delayed proliferation via Fc-dependent mechanisms, presumably ADCC.

## DISCUSSION

In this report, we described the characterization and development of 2448, a novel mAb that was generated by whole cell immunization of hESC, but was also shown to recognize ovarian and breast cancer cells. Unlike previous hESC-generated mAbs from our lab, 2448 was shown to target a unique glycosylated epitope on annexin A2 (ANXA2; also known as lipocortin II or calpactin-1 heavy chain), a calcium-dependent phospholipid binding glycoprotein belonging to the annexin family of proteins. To the best of our knowledge, ANXA2 was not previously studied as a surface marker on hESC. Nonetheless, its presence in hESC and induced pluripotent stem cells was reported by Munoz *et al.* in a proteome study of pluripotent stem cells [19]. Furthermore, as a progastrin receptor, ANXA2 may be involved in stem cell-related NF-κB and β-catenin signaling pathways [20]. Alternatively, ANXA2 was previously identified as a potential therapeutic target in cancer. Upregulated expression of ANXA2 was observed in multiple cancers including epithelial ovarian cancer [21, 22] and breast cancer [23, 24]. As a multifunctional protein, ANXA2 was reported to have a role in tumor cell adhesion, migration and invasion, proliferation, neovascularization and angiogenesis and membrane trafficking [25–29].

Nonetheless, anti-ANXA2 antibody therapies have yet to reach clinical trials. One reason for this may be due to the difficulty in generating an antibody against a suitable surface epitope on ANXA2. Although extracellular ANXA2 was reported in diverse cell types including endothelial and tumor cells, surface epitopes on ANXA2 may be challenging to recapitulate (for antibody generation purposes). ANXA2 notably lacks a transmembrane domain and a classical signal peptide sequence (see Supplementary Figure 1). And instead, several alternative mechanisms of surface translocation have been proposed including post-translational modifications such as phosphorylation [29] and glycosylation [30, 31].

As 2448 was generated by whole-cell immunization, it was not surprising that 2448 targeted a glycan epitope on the cell surface [7]. ANXA2 has multiple potential N-linked and O-linked glycosylation sites (Supplementary Figure 1B) and glycoforms of ANXA2 have been reported in multiple cell types including cancer [15, 31]. The ability of 2448 to lose binding after periodate treatment further provides evidence that extensive glycan branching is present on ANXA2. So far, other anti-ANXA2 mAbs have yet to demonstrate evidence of binding to glycan epitopes.

The ability of 2448 to target glycan epitopes on ANXA2 may also account for its specificity towards an epithelial (versus mesenchymal) phenotype of EMT. ANXA2 has been reported to play a possible role in cancer-related EMT [32–34]. For example, knockdown of ANXA2 in breast cancer cells resulted in a more epithelial phenotype, in which cells were unable to undergo EGF-induced EMT [33]. And downregulation of a phosphorylated ANXA2 correlated with loss of ability in pancreatic cancer cells to undergo TGFβ-Rho mediated EMT [29]. Unlike these reports, our data suggested that surface expression of a glycoform of ANXA2 is uniquely present on cancer cells prior EMT transformation. These conflicting reports provide further evidence that the role of ANXA2 in cancer-related EMT has yet to be fully elucidated. Importantly, these studies did not investigate glycoforms of ANXA2. Proteins associated with cell adhesion (eg. beta-catenin, cytokeratins, E-cadherin) are often closely associated with glycosylation changes during EMT [35, 36]. Therefore, ANXA2 may undergo similar glycosylation changes during EMT.

As glycan epitopes of ANXA2 have not been studied previously, we went on to demonstrate that the unique epitope of 2448 represented a functional target. Previous studies on ANXA2 have attempted to neutralize its role in cell to cell adhesion and promoting tumor cell invasion and angiogenesis [37, 25, 24, 29]. For example, an anti-ANXA2 mAb reduced ovarian cancer cell invasion using a chick chorioallantoic membrane assay [25]. An anti-ANXA2 mAb also inhibited neoangiogenesis in a mammary fat-pad xenograft model via reduction of localized plasmin generation and the activation of MMP-2/9 [24]. Finally, anti-ANXA2 mAbs significantly inhibited EGF-induced proliferation and metastasis by successfully blocking of EGFR homo-dimerization, phosphorylation and internalization in breast cancer [37]. Unlike these anti-ANXA2 mAbs that were reported in the cancer literature, 2448 was not specifically tested for these activities; however, no effect was observed in cell proliferation and migration assays *in vitro* (Supplementary Figure 6).

Instead, 2448 was chimerized (ch2448) and developed as an ADC and as an afucosylated mAb with potent ADCC activity. As both 2448 and ch2448 rapidly internalized into cells and deliver secondary saporin conjugates, ch2448 was directly conjugated to saporin (ch2448-saporin) and evaluated. Potent cytotoxicity was demonstrated with IC_50_ values in the nanomolar range for both IGROV1 and SKOV3 cells. Similar IC_50_ value was observed with clinically-approved trastuzumab emtansine (T-DM1), which had IC_50_ values in the sub-nanomolar range (e.g. 0.04 nM for HER2-positive BT474 cells [38]). Other pre-clinically-evaluated ADCs had similar IC_50_ values in the sub-nanomolar range [16, 39]. Despite these promising *in vitro* data, the saporin-based ADCs are not ideal therapeutics. As a proof-of-concept, the findings here suggested that ch2448 could be engineered and further optimized as an ADC with different linkers and payloads such as MMAE (monomethyl auristatin E) or DM1 (mertansine). The next step would be to optimize ch2448 as an ADC for *in vivo* testing.

Apart from developing ch2448 as an ADC, the ability of ch2448 to elicit ADCC activity was assessed. ADCC activity is an important feature for the clinical efficacy of mAbs therapies and recent studies have shown that afucosylation can enhance the ability of therapeutic IgGs to elicit ADCC. To date, afucosylated anti-CD20 and anti-CCR4 mAbs have been approved against hematological cancers and preclinical studies of afucosylated mAbs against solid tumors have also shown promising results [40–44]. Similar to reported afucosylated mAbs that have been reported in the literature, aF-ch2448 exhibited superior ADCC activity compared to ch2448. A 3 to10-fold increase in ADCC activity was reported for afucosylated anti-CD20 mAb [41] and up to a 10–11 fold increase in afucosylated anti-EGFR and anti-HER mAbs [42]. In comparison, aF-ch2448 demonstrated up to 26-fold better activity compared to (wildtype) ch2448. Importantly, this increase in ADCC activity applied to the weak binding SKOV3 cell line, suggesting that this approach could expand the anti-tumor efficacy of 2448.

To assess for potential anti-tumor activity of ch2448 i*n vivo*, IGROV1 subcutaneous tumor xenograft models were used. Treatment with ch2448 as a full length IgG1but not as a F(ab’)2 inhibited subcutaneous tumor cell growth. Therefore, *in vivo* tumor suppression by ch2448 relied on the capacity of ch2448 to interact with Fc receptors on mouse effector cells (which include NK cells, macrophages and complement proteins). Other anti-ANXA2 mAbs demonstrated the ability to reduce tumor burden in ovarian [25], breast [45] and glioma [46] cancer mouse models. But as previously mentioned, these studies focused on inhibiting the functional roles of ANXA2, especially its role as an activator of plasmin generation in metastasis and angiogenesis. The subcutaneous xenograft model employed in this study limited the possibility that ch2448 was regulating plasmin activation [47]. In addition, no metastasis or angiogenesis was observed. Therefore, these findings reflect the ADCC activity of ch2448 that was demonstrated *in vivo*.

It is also important to note that the animal studies present here were not ideal for studying ADCC activity ch2448 due to weaker interactions between activating mouse Fc receptors and the human Fc region of an IgG. Nonetheless, in both *in vitro* and *in vivo* studies, others have demonstrated that human IgG_1_ can interact with mouse Fc receptors and induce ADCC or ADCP activity with mouse NK cells or mouse macrophages [48, 49]. As the effect would be less than human Fc and human Fc-receptors, the effect of ch2448 to elicit ADCC could be even more pronounced in mouse models with humanized immune systems [50].

In summary, the novel IgG_1,_ 2448, was shown to target a unique glycosylated surface epitope on ANXA2. Expression was conserved across hESC and ovarian and breast cancer cells expressing an epithelial phenotype of EMT. As a possible therapeutic candidate for ovarian and breast cancer, 2448 demonstrated anti-tumor activity via two independent mechanisms of action: intracellular delivery of a toxin as an ADC (ch2448-saporin) and ADCC as an afucosylated ch2448 (aF-ch2448). Good tumor suppression was observed using ch2448 in a subcutaneous tumor xenograft mouse model. Overall, the findings in this study constitute strong preclinical evidence for further development of 2448 as a targeted therapy.

## MATERIALS AND METHODS

### Cell culture

The human ES cell line, HES-3 (46 X, X), was obtained from ES Cell International and cultured as described previously [5]. Ovarian cell lines were a gift from the lab of Dr. Ruby Huang (Cancer Science Institute, Singapore). The breast carcinoma cells, MCF7-D10 and MCF7-2101, were a gift from the lab of Dr. Jean Paul Thiery (Institute of Molecular and Cell Biology, Singapore). Other breast cell lines were obtained from the American Type Culture Collection (ATCC) and the National Cancer Institute 60 (NCI-60) panel of cancer cell lines. Cells were cultured at 37° C in a humidified incubator with 5% CO_2_ at low passage numbers (<40 for *in vitro* studies and <20 for *in vivo* studies). Standard culture media was used as recommended by ATCC and stored in freezing medium of 90% FBS (GE Healthcare) and 10% DMSO (Sigma Aldrich). Cell counting was done based by the Trypan Blue dye (Sigma Aldrich) exclusion method or by NucleoCounter (ChemoMetec) as per the manufacturer’s instructions.

### Antibody generation and purification

Antibody 2448 was generated by a mouse hybridoma approach using live intact HES-3 as the immunogen [5]. Hybridomas were maintained in ClonaCell™-HY Medium E (Stem Cells Technologies) at 37° C in a humidified incubator with 5% CO_2_. Chimerization was done in-house by the Animal Cell Technology group at BTI [15] and expressed in DG44-CHO cells. Cultures were maintained in BTI’s proprietary serum-free media.

Purification was done using the ÄKTA Explorer 100 (GE Healthcare) system. Cultured supernatants were subjected to Protein A chromatography (Tosoh; Toyopearl AF-rProtein A-650F) and ion exchange chromatography (Bio-Rad; UNOsphere^™^ Q). Purified products were evaluated on a Superdex200 PC 3.2/30 column (GE Healthcare) using a high performance liquid chromatography system (Shimadzu). Antibodies were additionally analyzed by SDS-PAGE, and protein concentrations were determined by absorbance at *A*_280_ using Nanodrop 1000 (Thermo Fisher Scientific). For *in vivo* studies, endotoxin was measured to ensure levels were below 0.1 EU/mL using the Endosafe^®^ Endotoxin Testing System (Charles River).

The following antibodies were also generated by whole cell immunization of hESC and used as mouse isotype controls: IgG clone 8 [8]; IgM clone 63; IgG B11. The following mAbs were used as chimeric isotype controls: human IgG (Southern Biotech) and chimeric IgG chTNA2 and IgG chTNB1 (which were generated by whole cell immunization of BT549 cells).

### F(ab’)2 generation

The IdeS protease (Genovis; FabRICATOR) was used to generate F(ab’)2 fragments from whole IgG. Briefly, IdeS enzyme was added to purified antibody at 1 unit of enzyme per 1 μg of antibody in 50 mM HEPES/ 150 mM NaCl buffer solution. The mixture was incubated at 37° C for 1 h. Histadine-tagged IdeS enzyme was removed by Ni-NTA spin columns (Qiagen n). Removal of Fc was verified using a biotinylated anti-human Fc secondary antibody (Sigma Aldrich) and appropriate streptavidin conjugates (Dako) for detection by Western Blot analysis and flow cytometry.

### Flow cytometry

Cells were harvested as single-cell suspensions using trypsin (Thermo Fisher Scientific). Each sample of 1–2 × 10^5^ cells was thoroughly washed in 1% bovine serum albumin (BSA; Sigma Aldrich) in phosphate buffered saline (PBS) buffer (Thermo Fisher Scientific). Cells were incubated with primary mAb at 4° C for 45 min, washed and then incubated with the appropriate fluorescein isothiocyanate (FITC)-labeled secondary antibody (goat anti-human kappa light chain mAbs (Sigma Aldrich) or goat anti-mouse Ig polyclonal (Dako) for 15 min at 4° C. After incubation, cells were washed and sample data acquisition was done using a BD FACSCalibur™ (BD Biosciences) or Guava^®^ easyCyte (Millipore). Data analysis was done using FlowJo™ software v7.6.3 (Tree Star). Percentage of binding was determined using M-gating set at the 97th-98th-percentile based on the negative control.

### Immunocytochemistry

Cell cultures were fixed with 4% paraformaldehyde (Sigma Aldrich) and blocked with goat serum (Dako) in PBS for 1 h at room temperature. Primary incubation was done with 2448 (1:500) directly conjugated with DyLight™ 488 NHS Ester (Thermo Fisher Scientific) at 4° C. Cell nuclei were counterstained with 4′,6-Diamidino-2-Phenylindole, Dihydrochloride (DAPI; 1:1000, Thermo Fisher Scientific). Microscope images were taken using an Axiovert 200 (Zeiss).

### Gel electrophoresis and Western blot analysis

Cells were harvested by manual scraping and as required, enriched for membrane proteins via the Membrane Protein Extraction Kit (BioVision). Briefly, cells were re-suspended in homogenization buffer and centrifuged at 700 g for 10 min at 4° C. Supernatant was then aspirated and centrifuged at 10,000 g for 30 min at 4° C. Cell pellet of total membrane proteins was collected and lysed with buffer containing 2% Triton X-100 (Bio-Rad) in PBS which was supplemented with protease inhibitors (Calbiochem). Total protein concentration was determined using the *DC*™ Protein Assay (Bio-Rad).

For gel electrophoresis, samples were prepared with loading dye at a final concentration of 50 mM Tris-HCl, 2% SDS, 10% glycerol, 0.02% bromophenol blue. For reducing conditions, 2–5% beta-mercaptoethanol was added. Samples along with SeeBlue Plus2^®^ or Page2™ protein standards (Thermo Fisher Scientific) were subjugated to SDS-PAGE using a 4–12% Bis-Tris gradient gel (Thermo Fisher Scientific) and 1X MOPS buffer (Thermo Fisher Scientific). Samples were analyzed using Coomassie Brilliant Blue staining solution (Sigma Aldrich) or Western blotting.

For Western blotting, products from gel run were transferred to polyvinylidene difluoride (PVDF) membrane (Bio-Rad) and blocked with 5% non-fat milk or Odyssey^®^ Blocking Buffer (LI-COR) for 1 h. Primary mAb (mouse anti-annexin A2 mAb (BD Biosciences); mouse anti-annexin A2 mAb (Invitrogen); rabbit anti-annexin A2 pAb (Santa Cruz); mouse anti-E-Cadherin mAb (BD Biosciences); mouse anti-Vimentin mAb (Dako); mouse anti-human Fc-specific mAb (Sigma)) or biotinylated lectin (*Aleuria Aurantia* Lectin (Vector Labs)) incubation was done overnight at 4° C. Appropriate secondary horseradish peroxidase (HRP)-conjugated antibody (goat anti-mouse pAb (Dako); anti-human pAb (Dako)) or HRP-conjugated streptavidin (Dako), was done for 1–2 h and visualized upon addition of chemiluminescence substrate (GE Healthcare). Blot images were captured and developed by film (Roche) using the Medical X-ray Processor 2000 (Kodak) or the ChemiDoc™ Imaging System (Bio-Rad).

### Immunoprecipitation and mass spectrometry analysis

Immunoprecipitation (IP) was done to enrich for the antigen target of 2448. Lysate from IGROV1 cells were precleared with Protein G-Sepharose resin (GE Healthcare) and clarified by centrifugation. Samples were subsequently incubated overnight on a rotator with 2448-coated Protein G-Sepharose resin. After multiple washes with PBS and saline solution containing 150 mM NaCl at pH 7.4, elution was done with SDS-containing buffer. IP products were separated by SDS-PAGE and analyzed on Western blot or stained using SilverQuest kit (Invitrogen). Silver stained bands corresponding to the antigen of interest were manually excised and soaked overnight in 2.5 mM ammonium bicarbonate in 50% acetonitrile solution at 4° C. Samples were subjected to in-gel trypsin proteolysis and sent for liquid chromatography-tandem mass spectrometry (LC-MS/MS) analysis.

### Transient knockdown of annexin A2 (ANXA2)

IGROV1 cells were seeded in T175 flasks at 1 × 10^6^ cells and grown to 30–50% confluency. Transfection of a validated set of human ANXA2-specific siRNA (Thermo Fisher Scientific) or scramble siRNA control (Thermo Fisher Scientific) was done in serum-free media at 30 pM using Lipofectamine RNAiMAX (Thermo Fisher Scientific). Post 5 h incubation at 37° C, media was removed and replaced with fresh media containing 10% serum. Cells were harvested within 72 h for Western blot analysis. Densitometry was done using *IMageJ* software (National Institutes of Health) and normalized to beta-actin expression.

### Periodate treatment

Lysate from IGROV1 cells was separated by SDS-PAGE and transferred to PVDF membrane (Bio-Rad). Subsequently, blots were washed with sodium acetate buffer (100 mM at pH 4.5; Merck Millipore), and incubated with sodium *meta*-periodate (100 mM, Sigma-Aldrich) for 30 min in the dark. Blots were subsequently washed with sodium acetate buffer, PBS and quenched with 0.5 M of sodium borohydride (Sigma) for 30 min. Control blots were similarly incubated but without the addition of sodium *meta*-periodate. Blots were incubated in Odyssey^®^ Blocking Buffer (LI-COR) for 30 min and incubated with primary mAb and secondary detection Ab as previously described.

### Antibody internalization assay

Antibodies were lysine conjugated to the CypHER5E mono NHS ester dye (GE Healthcare) according to the manufacturer’s protocol. IGROV1 cells were harvested and incubated in the presence of sodium azide (Sigma #S8032) at 0.1% w/v in 1% BSA/PBS or buffer alone for 30 min at 4° C. Dye-conjugated mAb or free-dye control was then added to cells with or without sodium azide. Cells were transferred to 37° C to induce internalization, and fluorescence was measured on the BD FACSAria™ II (BD Biosciences) flow cytometer. The mean fluorescence intensity (MFI) of internalized dye was analyzed using FlowJo™ software v7.6.3 (Tree Star).

### Antibody drug conjugate (ADC) assay with secondary saporin conjugates

Cells were seeded in 96-well plates (Corning) at 1000 or 2000 cells per well. Primary antibody, 2448 or ch2448 (10 µg/mL) was pre-mixed with appropriate secondary saporin conjugate, mAb-ZAP or HUM-ZAP (Advanced Targeting Systems) at a 1:3 molar ratio for 15 min at room temperature. At 24 h post-cell seeding, pre-m, primary mAb, secondary conjugate or buffer alone was added to wells. At 72 h post-treatment, viable (metabolically active) cells were measured based on the presence of ATP, using the CellTiter-Glo^®^ Luminescent Cell Viability Assay kit (Promega). Data were expressed as a percentage of the buffer-treated control.

### Antibody drug conjugate (ADC) assay with ch2448-saporin conjugate

Direct conjugation of saporin to antibodies was outsourced to Advanced Targeting Systems (ATS). Drug to antibody molar ratios of ch2448-saporin, chTNA2-saporin and human Ig-saporin were 2.5, 2.9 and 3.1, respectively. Cytotoxicity of ch2448-saporin was evaluated on IGROV1, SKOV3 and IOSE523 cell lines. Cells were seeded in 96-well plates (Corning) at 1000 cells per well in 90 μl of media. The following day, ch2448-saporin and chTNA2-saporin were serially diluted and 10 μl of each dilution was added to wells. As a control, free saporin and ch2448 were added in a separate set of plates. Post 72 h incubation, the cell viability was measured using the CellTiter-Glo^®^ Luminescent Cell Viability Assay kit (Promega) according to the manufacturer›s instructions. Data are expressed as the % control, measuring the viability of treated cells with that of untreated cells. Dose response curves and IC_50_ values were calculated using GraphPad Prism 6 (GraphPad).

### Antibody-dependent cell-mediated cytotoxicity (ADCC) assay

ADCC activity was measured using the ADCC Reporter Bioassay (Promega). Briefly, cells were seeded at 5,000 cells per well in a 96-well clear bottom black tissue culture plates (Corning) in low 4% IgG-serum (Promega) media. Serial dilutions of primary antibody were incubated in triplicate wells for approximately 15 min at 37° C, 5% CO2. Following incubation, engineered effector cells were added to the wells at approximately 150,000 cells per well. After 5 to 16 h (as indicated in results), Bio-Glo™ Luciferase Assay Substrate (Promega) was added to the wells and luminescence was measured using the Infinite^®^ 200 microplate reader (Tecan). Estimated EC_50_ values were calculated by linear regression analysis using GraphPad Prism 6 (GraphPad).

### Tumor xenograft mouse models

Four- to six-week-old female BALB/c nude mice (InVivos) were used in this study. On day 0, mice were inoculated with a total of 5 × 10^6^ IGROV1 cells in 100 μl of cell media and high concentration matrigel (BD Biosciences) at a 1:1 dilution volume. For the biodistribution study: antibodies ch2448 and IgG control were labelled with a near infrared fluorescent (NIR) dye CF750 using the XenoLight CF750 rapid antibody-labelling kit (Caliper Life Sciences). When tumor volume reached 300–400 mm^3^ (on week 10), dye-conjugated ch2448 and control conjugate were administered by intraperitoneal injection at 100 μg per 100 μl of buffer (50 mM HEPES, 150 mM NaCl) per mouse. At 72 h and 94 h post injection, mice were anesthetized with 2–3% isoflurane and imaged using the IVIS^®^ Spectrum imaging system (Caliper Life Sciences). Data was analyzed using the Living Image software 3.2 (Caliper Life Sciences). For the antibody efficacy study: post-24 h cell inoculation, antibody (or F(ab’)2) was given via intraperitoneal injection at 100 μl volumes at 5 mg/mL in 50 mM HEPES, 150 mM NaCl buffer. Subsequent administration was done weekly as indicated. Size of the primary tumor was measured weekly by digital calipers.

Tumor volume (TV) was calculated based on the formula: TV = ((L × L × W)/2), where W (width) and L (length) are the short and long diameter, respectively Statistical significance was measured by a two-sided unpaired Student’s *t*-test (^*^p < 0.05; ^**^p < 0.01; and ^***^p < 0.001). Mice were euthanized before tumor size reached >2500 mm^3^ or when persistent side effects (e.g. swollen lymph nodes or drastic body weight loss) were observed over a period of more than two weeks. Euthanization was done by CO_2_ inhalation followed by cervical dislocation. Animals were handled according to A*STAR (BRC) IACUC Protocol No.:151001, in accordance with the National Advisory Committee For Laboratory Animal Research (NACLAR) Guidelines.

## SUPPLEMENTARY MATERIALS FIGURES, TABLE AND VIDEO




