# Supplementary material for: Targeting of embryonic annexin A2 expressed on ovarian and breast cancer by the novel monoclonal antibody 2448
Source: Oncotarget. 2018 Jan 10;9(17):13206–21. doi: 10.18632/oncotarget.24152 (PMC5862572; doi:10.18632/oncotarget.24152)
Supplement: Supplementary file 1 [file oncotarget-09-13206-s001.pdf]

Targeting of embryonic annexin A2 expressed on ovarian and breast cancer by the novel monoclonal antibody 2448

SUPPLEMENTARY MATERIALS

Cell lines

HES-3 was used to form embryoid bodies (EB) as described in (5). 2102Ep was a gift from the lab of Prof. Peter Andrews (University of Sheffield) and was cultured as described in (9). HEK-293, HFF, hTERT-HME1, hTERT-HPNE and IMR-90 were obtained from the American Type Culture Collection (ATCC) and cultured in standard media as recommended by ATCC. InEpC was purchased from Lonza and cultured in standard media as recommended by Lonza. Cells were stored in freezing medium of 90% FBS (GE Healthcare) and 10% DMSO (Sigma Aldrich).

Immunohistochemistry

The human ovarian tissue microarray (TMA) OVC481 (Pantomics) and breast TMA BRC961 (Biomax) were obtained and stained with antibody 2448 or mouse isotype control (Invitrogen). After dewaxing and rehydration, antigen retrieval was done using TRIS-Buffer (Invitrogen) at pH 9 for 30 min at 95º C. Endogenous peroxidase activity was blocked with 1% H202 (Millipore) in PBS for 30 min at room temperature. Slides were then blocked using goat serum (Invitrogen) in PBS for 1 hr. Primary mAb incubation (1:40) was done overnight and developed using the EnVision + System HRP (DAB) Kit (Dako). Slides were counterstained with haematoxylin (Sigma Aldrich), dehydrated with ethanol and cleared using Histo-Clear (National Diagnostics). Slides were mounted using medium containing limonene (Sigma Aldrich) and imaged on an Axio Scan. Z1 (Zeiss) slide scanner system. Staining method was validated on cell-line arrays using cell lines positive and negative for 2448 binding by flow cytometry.

Real-time monitoring of 2448 internalization

Antibodies were labeled with pH-RODO (Thermo Fisher Scientific) according to the manufacturer's protocol. Antibody was added to cell culture and visualized using DeltaVision Microscopy Imaging Systems (GE Healthcare).

Real-time monitoring of ch2448 and ch2448-saporin on cell growth

Cell growth was continuously monitored over time by cell impedance measurements using the xCelligence® real-time cell analyzer (Roche). Briefly, cell culture media was first loaded onto the 96-well E-plate to measure background impedance. IGROV1, IOSE523 and SKOV3 cells were plated at 1,000 cells per well and allowed to grow overnight in normal cell culture conditions. Cells were treated with antibody (ch2448) or antibody conjugates (ch2448-saporin or human IgG-saporin) at the beginning of the log phase of cell growth. Control wells were treated with buffer alone. All experiments were done in at least 5 wells per treatment condition. The outermost wells of the plate were not used. For dose response curves, IGROV1 were treated with dilutions of ch2448-saporin. The cells were monitored for growth until cells reached a death phase. Cell indices of wells were normalized after antibody treatment. The IC_{50} value on IGROV1 cells was determined when cells treated at the lowest concentration were reaching a stationary phase of cell growth. The IC_{50} value was calculated using the accompanying real-time cell analysis software (Roche).

Supplementary Table 1: Reactivity of 2448 on embryonic cells and normal adult cell

Cell line description		Binding
**Category**	**Name**	**2448**
**Embryonal Carcinoma**	**2102Ep**	++
**hESC**	**HES-3**	+++
	**EB**	-
**Human embryonal kidney**	**HEK-293**	-
**Human fetal lung**	**IMR-90**	-
**Human foreskin fibroblast**	**HFF**	-
**Human intestinal epithelial**	**InEpC**	-
**Human mammary epithelial**	**hTERT-HME1**	–/+
**Human pancreatic nestin-expressing**	**hTERT-HPNE**	–/+

Supernatant from hybridoma clones of 2448 was screened by flow cytometry on human embryonic stem cells and adult normal cells. Binding was observed on embryonal carcinoma and hESC lines but not on normal adult epithelial cells. Binding intensity was graded according to binding percentages after marker gating with a 2.0–2.5% cut-off from the negative control: "–" < 10% binding, "+" = 10–40% binding, "++" = 40–60%, "+++" = 60–80% and "++++" > 80%.

A

Annexin A2 Isoform 1 (P07355-1)

```
     10         20         30         40         50
MSTVHEILCK LSLEGDHSTP PSAYGSVKAY TNFDAERDAL NIETAIKTKG
     60         70         80         90        100
VDEVTIVNIL TNRSNAQRQD IAFAYQRRTK KELASALKSA LSGHLETVIL
    110        120        130        140        150
GLLKTPAQYD ASELKASMKG LGTDEDSLIE IICSRTNQEL QEINRVYKEM
    160        170        180        190        200
YKTDLEKDII SDTSGDFRKL MVALAKGRRA EDGSVIDYEL IDQDARDLYD
    210        220        230        240        250
AGVKRKGTDV PKWISIMTER SVPHLQKVFD RYKSYSPYDM LESIRKEVKG
    260        270        280        290        300
DLENAFLNLV QCIQNKPLYF ADRLYDSMKG KGTRDKVLIR IMVSRSEVDM
    310        320        330
LKIRSEFKRK YGKSLYYYIQ QDTKGDYQKA LLYLCGGDD
```

B

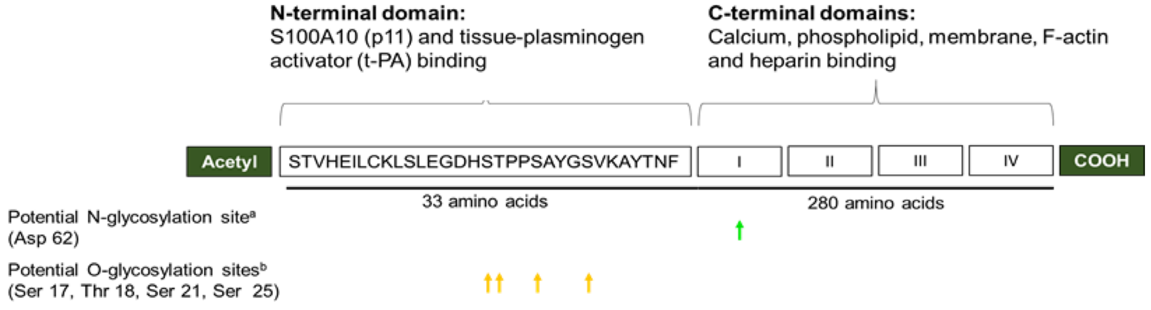

Supplementary Figure 1: Potential glycosylation sites of Annexin A2. (**A**) Amino acid sequence of Annexin A2 (isoform 1) from UniProt database. (**B**) Schematic of ANXA2 amino acid sequence and predicted N- and O-glycosylation sites. [a]N-glycosylation sites (↑) were identified in silico by NetNGlyc 1.0 (with the sequence Asn-Xaa-Ser/Thr) [b]O-glycosylation sites (↑) were identified by NetOGlyc 4.0 (with sequence GalNAC-α-Ser/Thr).

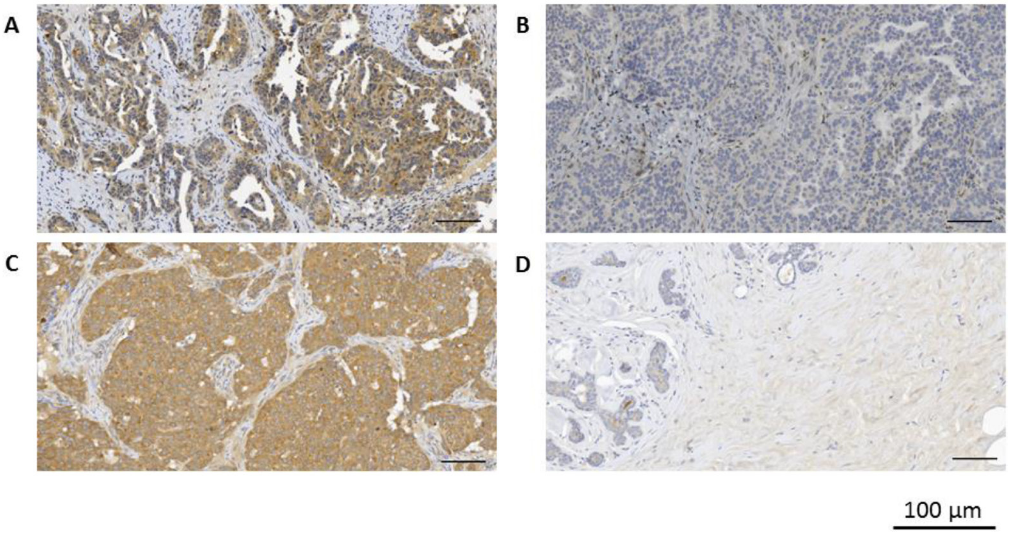

Supplementary Figure 2: Binding of antibody 2448 on ovarian and breast tumor tissue. Antibody 2448 was stained on ovarian and breast multi-tumor tissue microarrays. Representative images of strong binding of 2448 were shown on (**A**) serous cystadenocarcinoma (Grade II Stage, T1N0M0 at Position B2) tissue and not on (**B**) uninvolved adjacent ovary tissue. Similarly, binding was shown on (**C**) invasive ductal carcinoma (Grade II Stage, T3N2M0 at Position C11) tissue and not on (**D**) normal breast tissue.

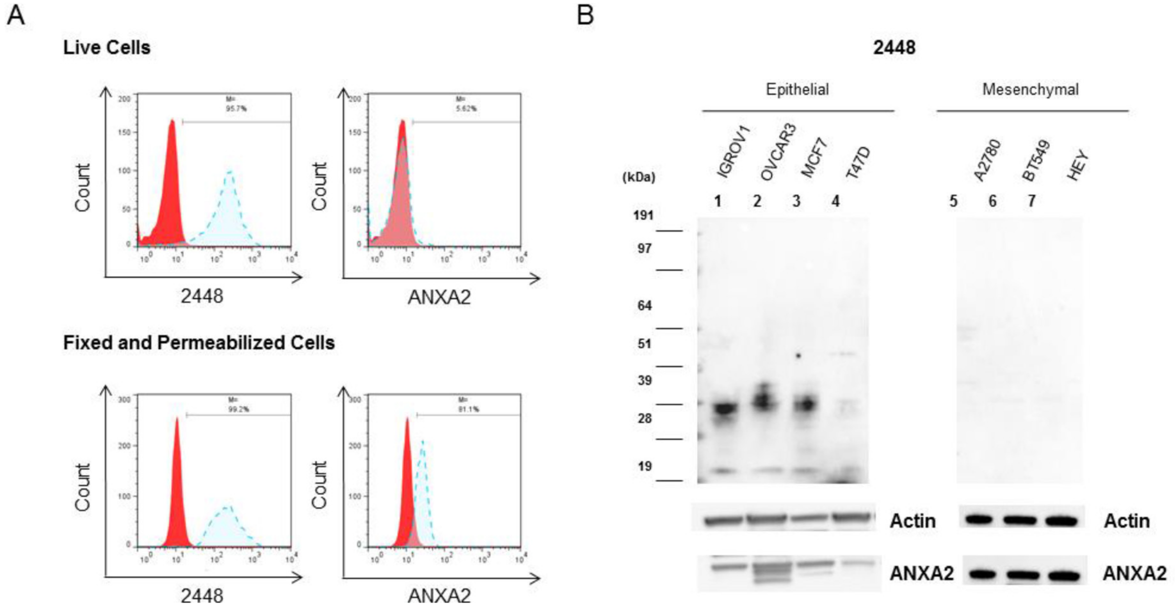

Supplementary Figure 3: Antibody 2448 targets a unique epitope on annexin A2 (ANXA2). (**A**) Surface binding of 2448 on IGROV1 cells was measured by flow cytometry. Antibody 2448 demonstrated binding on live cells compared to a commercial anti-ANXA2 mAb (as indicated by a shift in dashed-lined histograms). Binding of the commercial mAb was only observed when cells were fixed and permeabilized. Results showed that unlike 2448, the commercial mAb was not able to target surface epitopes of ANXA2. (**B**) The expression of ANXA2 on membrane-protein (MP) enriched lysate was analyzed by Western Blot. ANXA2 is present in all reported cell lines as depicted by the antigen expression of a commercial anti-ANXA2 mAb. Antigen expression of 2448 was only present on cells with an epithelial phenotype of EMT. Beta-actin expression was used as a control.

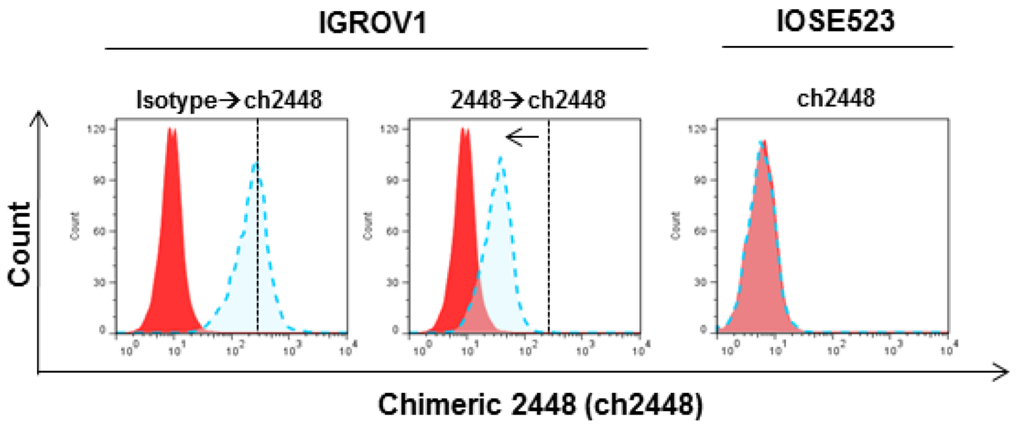

Supplementary Figure 4: Characterization of chimeric 2448 (ch2448). The chimeric variant of 2448 (ch2448) retained binding specificity of parental (mouse) 2448. IGROV1 cells were pre-incubated with 2448 (or non-binding isotype control) followed by ch2448. Binding of ch2448 was inhibited by 2448 (middle panel) but not the isotype control (left panel). Like 2448, ch2448 did not bind on the IOSE523 (right panel).

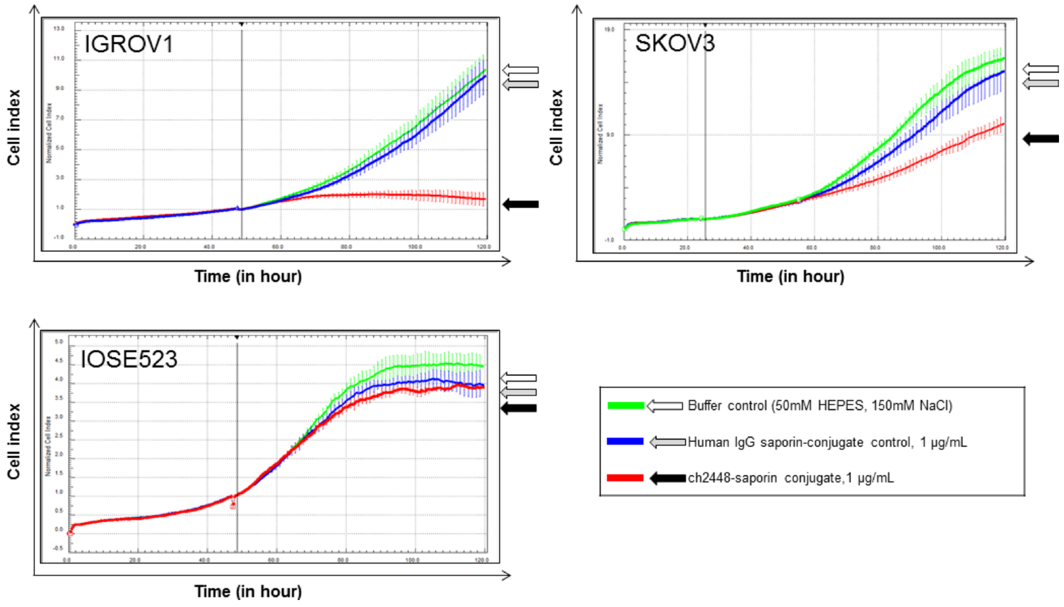

Supplementary Figure 5: Real time-monitoring of ch2448-saporin on cell proliferation. IGROV1, IOSE523 and SKOV3 cells were incubated with ch2448-saporin, human IgG-saporin control or HEPES buffer as a vehicle control. Cell proliferation of treated and control cultures were subsequently monitored using cell impedance readings via the xCelligence® real-time cell analyzer (Roche). The ch2448-saporin conjugate demonstrated sustained cytotoxicity on 2448-positive IGROV1 cell line but not on the 2448-negative IOSE523 cell line. Lower cytotoxicity was demonstrated on the SKOV3 cell line.

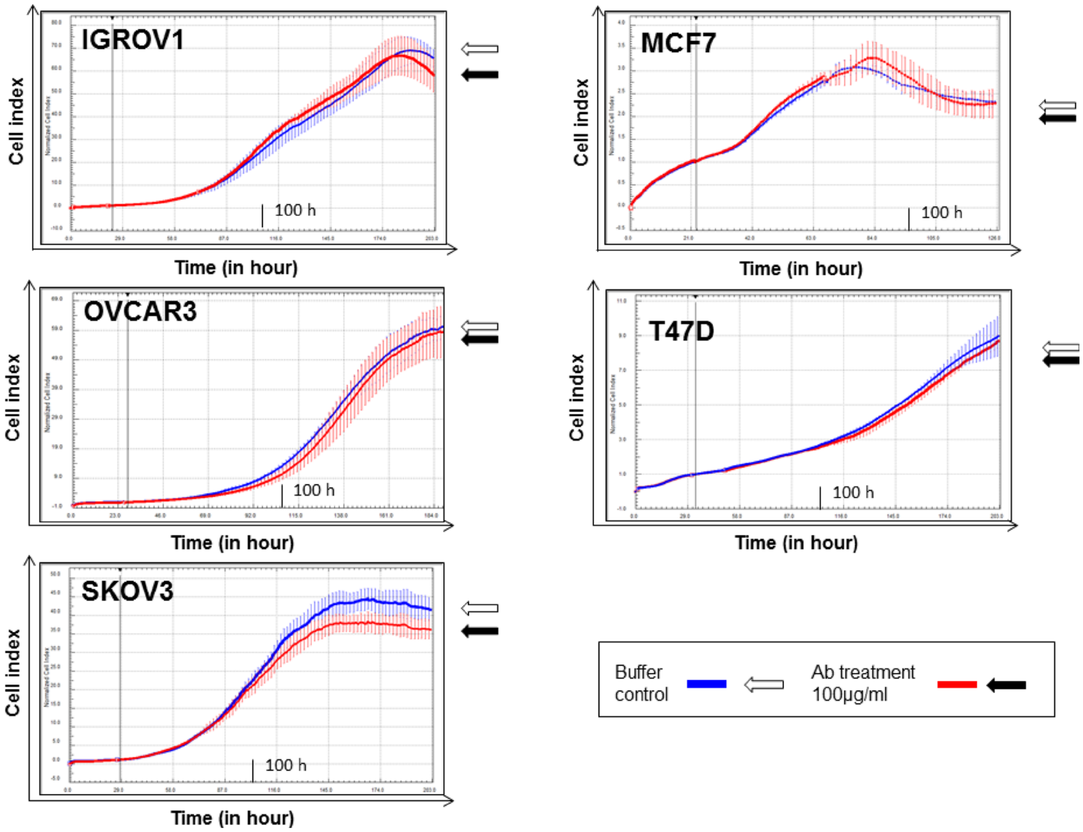

Supplementary Figure 6: Real-time monitoring of 2448 on cell proliferation. IGROV1, MCF7, OVCAR3, SKOV3 and T47D cells were incubated with ch2448 or HEPES buffer as a vehicle control. Cell proliferation of treated and control cultures were subsequently monitored using cell impedance readings via the xCelligence® real-time cell analyzer (Roche). No anti-proliferative effect was detected with treatment of ch2448.

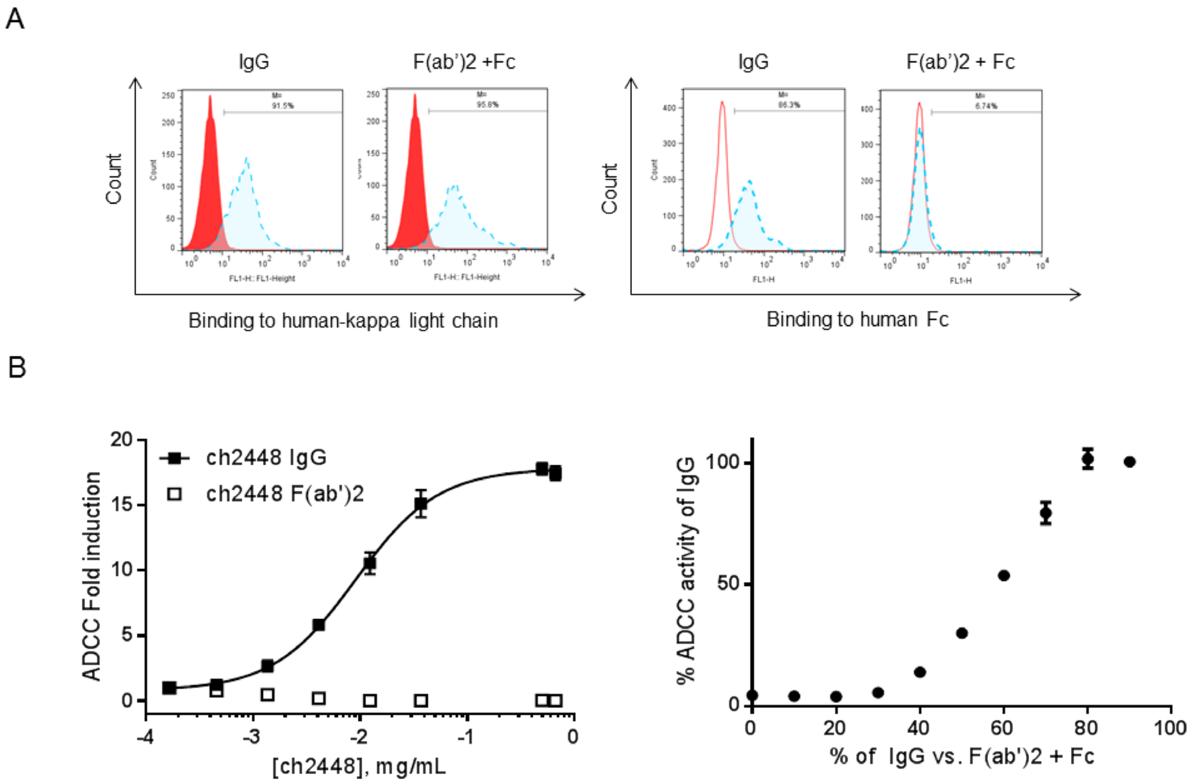

Supplementary Figure 7: Characterization of ch2448 as a F(ab')2. Binding and ADCC activity of F(ab')2 fragments of ch2448 (**A**) The F(ab')2 fragment of ch2448 retained binding as a full-length IgG on IGROV1 cells. Samples were first incubated in primary antibody, followed by a FITC-conjugated anti-human kappa light chain secondary antibody (as indicated by a shift in dashed-lined histograms). Removal of Fc was confirmed by no binding to an anti-human Fc secondary antibody. (**B**) F(ab')2 fragment of ch2448 did not elicit ADCC activity compared to (ch2448) IgG control. For combination of IgG and fragments of ch2448, the level of ADCC activity was determined by the ratio of IgG to F(ab')2 + Fc fragments. Antibody ch2448 elicited a 15-fold increase in ADCC activity.

Supplementary Video 1: Internalization of 2448. Cancer cells were pre-incubated with 2448 conjugated to a pH-sensitive dye at 4° C (*t* = 0 min). Subsequently, samples were incubated at 37° C. Antibody-internalization was visualized by time-lapse fluorescence microscopy. Internalization was observed as fluorescence signal within a 24 h period.
